# Glycyrrhizic Acid Promotes M1 Macrophage Polarization in Murine Bone Marrow-Derived Macrophages Associated with the Activation of JNK and NF-*κ*B

**DOI:** 10.1155/2015/372931

**Published:** 2015-11-19

**Authors:** Yulong Mao, Baikui Wang, Xin Xu, Wei Du, Weifen Li, Youming Wang

**Affiliations:** Key Laboratory of Animal Molecular Nutrition of Education of Ministry, College of Animal Sciences, Zhejiang University, 866 Yu Hang Tang Road, Hangzhou, Zhejiang 310058, China

## Abstract

The roots and rhizomes of* Glycyrrhiza* species (licorice) have been widely used as natural sweeteners and herbal medicines. The aim of this study is to investigate the effect of glycyrrhizic acid (GA) from licorice on macrophage polarization. Both phenotypic and functional activities of murine bone marrow-derived macrophages (BMDMs) treated by GA were assessed. Our results showed that GA obviously increased the cell surface expression of CD80, CD86, and MHCII molecules. Meanwhile, GA upregulated the expression of CCR7 and the production of TNF-*α*, IL-12, IL-6, and NO (the markers of classically activated (M1) macrophages), whereas it downregulated the expression of MR, Ym1, and Arg1 (the markers of alternatively activated (M2) macrophage). The functional tests showed that GA dramatically enhanced the uptake of FITC-dextran and* E. coli* K88 by BMDMs and decreased the intracellular survival of* E. coli* K88 and* S. typhimurium*. Moreover, we demonstrated that JNK and NF-*κ*B activation are required for GA-induced NO and M1-related cytokines production, while ERK1/2 pathway exhibits a regulatory effect via induction of IL-10. Together, these findings indicated that GA promoted polarization of M1 macrophages and enhanced its phagocytosis and bactericidal capacity. The results expanded our knowledge about the role of GA in macrophage polarization.

## 1. Introduction

Licorice, the root of* Glycyrrhiza uralensis*, is a well-recognized, natural sweetener and used as a traditional herbal medicine for the treatment of various pathological conditions, including allergies, liver disease, gastric ulcers, and adrenal insufficiency [1, 2]. A number of components have been isolated from licorice, including triterpene saponins, flavonoids, polysaccharides, and other substances [3]. Glycyrrhizic acid (GA), a major biologically active constituent of licorice root accounting for the sweet taste, is a triterpene glycoside containing one molecule of 18-glycyrrhetinic acid and two molecules of glucuronic acid [4]. So far, GA has been reported to have a variety of pharmacological activities like antiviral, antitumor, anti-inflammatory, and antioxidative activities [5–8]. It has been shown that GA can promote function of endothelial system and secretion of cytokines such as interleukin- (IL-) 1 and interferon- (IFN-) *α* [9], induce maturation of dendritic cells (DCs) [10], increase T cells proliferation and production of IL-2 and IFN-*γ* [11, 12], augment natural killer (NK) cell activity [13], enhance phagocytic capacity and nitric oxide (NO) production in activated macrophages [14], and downregulate the production of IL-8 and eotaxin-1 in human lung fibroblast cells [4]. These studies indicated that GA may serve as an immune modulator which precisely regulates the cellular immunity.

Macrophages have long been considered as important effector cells that play a key role in host defense and homeostasis [15, 16]. Depending on the microenvironment, macrophages can acquire distinct morphological and functional properties. Two extremes in the spectrum of macrophage phenotypes are often referred to as classically activated macrophages (or M1) and alternatively activated macrophages (or M2) [17, 18]. The M1 phenotype is polarized by Th1 cytokines such as IFN-*γ* and is characterized by high capacity to present antigen, high levels of inflammatory cytokines (TNF-*α*, IL-12, IL-6) secretion and increased levels of NO production, enhanced capacity to kill intracellular pathogens and tumor cells, and promotion of polarized Th1 and Th17 responses [15, 19, 20]. In contrast, M2 macrophages are polarized by Th2 cytokines such as IL-4 and IL-13 and are characterized by minimal production of inflammatory molecules and increased expression of mannose receptor (MR), chitinase-like Ym1, found in inflammatory zone-1 (FIZZ1), and arginase-1 (Arg1). M2 cells are associated with anti-inflammatory and homeostatic functions linked to wound healing, tissue remodeling and repair, scavenge debris, and participation in polarized Th2 reactions [21, 22]. Generally, M1 macrophages are considered proinflammatory cells, whereas M2 macrophages are anti-inflammatory.

However, there is limited information about how GA regulates the phenotype of macrophages. In the present study, we investigated the effect of GA on the polarization of murine bone marrow-derived macrophages (BMDMs) and found that GA promotes M1, rather than M2 macrophage polarization with enhanced phagocytosis and bacterial killing capacity. We further demonstrated that GA-mediated macrophage polarization involves activation of mitogen-activated protein kinases (MAPKs) and nuclear factor-*κ*B (NF-*κ*B) pathways.

## 2. Materials and Methods

### 2.1. Reagents

Dulbecco's modified eagle's medium (DMEM), LPS (*Escherichia coli* 0111:B4), FITC-dextran (40,000 Da), and glycyrrhizic acid (GA) were purchased from Sigma Chemical Co. (St. Louis, MO, USA). There was no detectable endotoxin (<0.10 endotoxin units/mL) in the GA samples, as determined by Endospecy. Recombinant moues IFN-*γ*, M-CSF, and IL-4 were obtained from PeproTech Inc. (Rocky Hill, NJ, USA). Anti-mouse antibodies FITC-F4/80, -CD80, -CD86, -MR and PE-MHCII, and -CCR7 as well as anti-NF-*κ*B p65 were obtained from Biolegend (San Diego, CA, USA). The ELISA kits for TNF-*α*, IL-6, IL-10, and IL-12p70 were obtained from eBioscience (San Diego, CA, USA). Antibodies against *β*-actin, iNOS, phospho-JNK, JNK, phospho-p38 MAPK, p38 MAPK, I*κ*B*α*, LaminB1, and HRP-conjugated anti-mouse and anti-goat IgG were purchased from Santa Cruz Biotech (Santa Cruz, CA, USA), and phospho-ERK1/2, anti-ERK1/2, phospho-STAT1, and anti-STAT1 antibodies were obtained from BD Pharmingen (San Jose, CA, USA). Inhibitors BAY 11-7082, SP600125, SB203580, and U0126 were purchased from Beyotime Biotechnology (Haimen, Jiangsu, China).

### 2.2. Animals

C57BL/6 mice (6~8 weeks old) were purchased from Slac Animal Inc. (Shanghai, China) and reared in Experimental Animal Center of Zhejiang University. All experimental protocols for animal studies were approved by the Institutional Animal Care and Use Committee of Zhejiang University.

### 2.3. Preparation of Bone Marrow-Derived Macrophages (BMDMs)

The preparation of BMDMs was modified from a previously described method [23]. Briefly, mice were killed by cervical dislocation and the femur and tibia of the hind legs were dissected and bone marrow cavities were flushed with 5 mL cold, sterile phosphate buffered saline (PBS). After lysing red blood cells, the bone marrow cells were washed, resuspended, and differentiated into BMDMs in DMEM with 10% FBS, 10 ng/mL M-CSF, 100 *μ*g/mL streptomycin, and 100 U/mL penicillin. Six days after initial BMDMs cell culture, the purity of F4/80^+^ cells was > 90%, as determined by flow cytometry (FACS).

### 2.4. Cytotoxicity Assay

Monolayers of BMDMs in 96-well microplate were cultured in DMEM supplemented with 10% FBS and incubated with GA (0~800 *μ*g/mL) for 48 h. The medium was replaced with fresh DMEM containing 0.5 mg/mL MTT. After 4 h of incubation, the supernatant was removed and the precipitation was dissolved with DMSO. Finally, the optical density was measured using SpectraMax M5 (MD) at OD_570_ [24].

Lactate dehydrogenase (LDH) release from damaged cells was determined 48 h after treatment with PBS (Blank control), GA (100 *μ*g/mL), IFN-*γ* (15 ng/mL) + LPS (15 ng/mL) (M1-positive control), and IL-4 (20 ng/mL) (M2-positive control). LDH activity in the culture supernatant was measured as previously described [25].

### 2.5. Flow Cytometry Analysis

To detect cell surface expression of CC chemokine receptor 7 (CCR7), MR, CD80, CD86, and MHC class II (MHCII), cells from different treatment groups were, respectively, collected and stained with antibodies against CCR7, MR, CD80, CD86, and MHCII for 30 min at 4°C and then washed twice with PBS and analyzed in a FACScalibur flow cytometer (Becton-Dickinson).

### 2.6. Total RNA Isolation and Real-Time PCR

Total RNA isolated from BMDMs (RNAiso plus, TAKARA) was reverse-transcribed using PrimeScript II 1st Strand cDNA Synthesis Kit (TAKARA). Real-time PCR was performed using SYBR Premix Ex Taq II (TAKARA) and the ABI 7500 real-time PCR system (Applied Biosystems). The following primers were used: TNF-*α* forward 5′-CCCTCACACTCAGATCATCTTCT-3′ and reverse 5′-GCTACGACGTGGGCTACAG-3′; IL-12p40 forward 5′-CCCATTCCTACTTCTCCCTCAA-3′ and reverse 5′-CCTCCTCTGTCTCCTTCATCTT-3′; IL-6 forward 5′-TAGTCCTTCCTACCCCAATTTCC-3′ and reverse 5′-TTGGTCCTTAGCCACTCCTTC-3′; Ym1 forward 5′-ACTCCTCAGAACCGTCAGAT-3′ and reverse 5′-GTAGCAGCCTTGGAATGCTTT-3′; MR forward 5′-GACGCTCTAAGTGCCATCTC-3′ and reverse 5′-ATAACTCTGTGCCCTTGATTCC-3′; FIZZ1 forward 5′-TCGTGGAGAATAAGGTCAAGGAA-3′ and reverse 5′-CGAGTAAGCACAGGCAGTTG-3′; iNOS forward 5′-CTCACCTACTTCCTGGACATTAC-3′ and reverse 5′-CAATCTCTGCCTATCCGTCTC-3′; Arg1 forward 5′-GCCTTTGTTGATGTCCCTAATGA-3′ and reverse 5′-CCACACTGACTCTTCCATTCTTC-3′; IL-10 forward 5′-GCTCTACTGACTGGCATGAG-3′ and reverse 5′-CGCAGCTCTAGGAGCATGT-3′; *β*-actin forward 5′-CGTTGACATCCGTAAAGACC-3′ and reverse 5′-AACAGTCCGCCTAGAAGCAC-3′. Fold changes were calculated after normalizing the change in expression of the gene of interest to the housekeeping gene *β*-actin using the threshold cycle values.

### 2.7. Cytokine Assay

Levels of TNF-*α*, IL-12p70, IL-6, and IL-10 in the culture supernatants were quantified using a sandwich ELISA kit (eBioscience) as per the manufacturer's instructions.

### 2.8. Western Blotting Analysis

Total cell lysates were prepared as previously described [26]. Nuclear and cytosolic extracts were fractionated using a Nuclear and Cytoplasmic Protein Extraction Kit (Beyotime Biotechnology) according to the manufacturer's instructions. Equal amounts of proteins from each sample were subjected to SDS-PAGE followed by transfer of proteins to nitrocellulose membranes. Membranes were blocked in no protein blocking solution (Sangon Biotech) and incubated with a primary antibody overnight at 4°C. After washing with TBST, membranes were incubated with secondary antibody linked to HRP. The blots were then developed with an ECL detection system as per the manufacturer's instructions.

### 2.9. Nitrite Generation Assay

Nitrite accumulation in culture supernatant was measured by the Griess method [27]. Cell-free culture supernatants were mixed with 100 *μ*L of 1% sulfanilamide, 0.1% N-(1-naphthyl-) ethyl-enediamine dihydrochloride, and 2.5% phosphoric acid. The absorbency was read within 5 min at 550 nm and actual concentration calculated using a standard curve with serial dilutions of sodium nitrite.

### 2.10. Phagocytosis Assay

To analyze the phagocytic activity of macrophages, BMDMs were pretreated with GA (100 *μ*g/mL) for 12 h and then incubated with FITC-dextran (1 mg/mL) at 37°C for 1 h. After incubation, the cells were washed twice with PBS and the percentage and mean fluorescence intensity (MFI) of intracellular FITC-dextran were determined by FACS.

### 2.11. Bacteria Killing Analysis

To determine the bactericidal function of macrophages, BMDMs were seeded at 2 × 10^5^ cells/well in 24-well culture plates (Corning) and pretreated with GA (100 *μ*g/mL) for 12 h, and then cells were washed and incubated with 1 × 10^7^ *Escherichia coli* (*E. coli*) (stain K88) or 2 × 10^7^ *Salmonella typhimurium* (*S. typhimurium*) (strain CMCC-50115) for 1 h at 37°C to allow bacterial adhesion and colonization. Thereafter, cells were thoroughly washed with PBS and incubated for 0 h or 24 h in DMEM containing gentamicin (25 *μ*g/mL). Finally, cell lysate from BMDMs containing intracellular bacteria was serially diluted with PBS and spread onto LB agar plates to determine bacterial viability.

### 2.12. Statistical Analysis

Results are presented as mean ± SD of three independent experiments. Statistical analyses were performed using two-tailed Student's *t*-test. Values of *P* < 0.05 were considered significant.

## 3. Results

### 3.1. Determination of the Noncytotoxic Dose of GA in BMDMs

**Figure 1:**
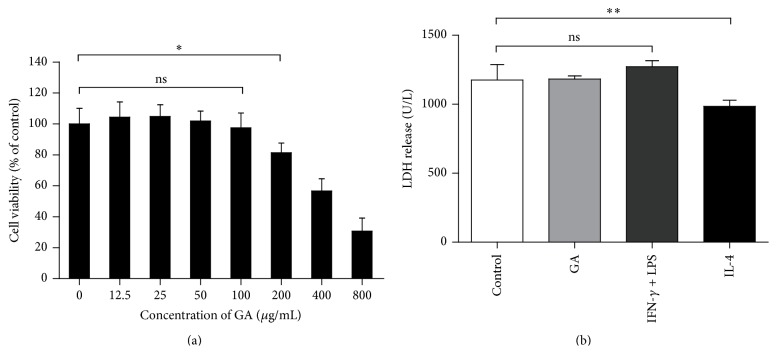
Determination of the noncytotoxic dose of GA. (a) BMDMs were incubated with GA at range from 0 to 800 *μ*g/mL for 48 h. Cell viability was determined by MTT method. The results are expressed as the percentage of viable cells and represent mean ± SD of eight samples. (b) Cell death was confirmed by measuring the release of the cytosolic marker LDH. BMDMs were treated with PBS, GA (100 *μ*g/mL), IFN-*γ* (15 ng/mL) + LPS (15 ng/mL), and IL-4 (20 ng/mL) for 48 h, and LDH activity in the supernatant was measured as described in Section 2. ^*∗*^ *P* < 0.05, ^*∗∗*^ *P* < 0.01 (*t*-test).

We evaluated the cytotoxicity of GA ranging from 12.5 to 800 *μ*g/mL on BMDMs and found the optimal viability was 100 *μ*g/mL, showing 97.44% survival (Figure 1(a)). Moreover, we confirmed cell damage of each treatment used in following experiments by measuring the release of the cytosolic marker LDH. Treatment with GA (100 *μ*g/mL) and IFN-*γ* (15 ng/mL) + LPS (15 ng/mL) for 48 h showed no significant difference of LDH release when compared with the control groups, while IL-4 (20 ng/mL) significantly decreased LDH release (Figure 1(b)). Therefore, 100 *μ*g/mL GA was used for the next experiments.

### 3.2. The Activation Profiles of BMDMs Treated with GA

**Figure 2:**
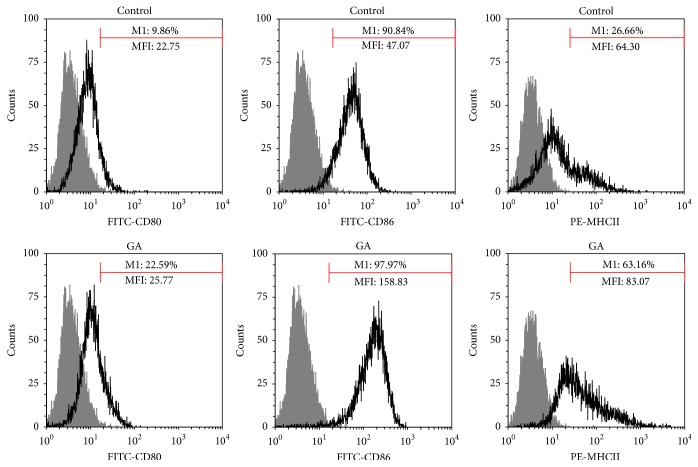
The activation profiles of BMDMs treated with GA. BMDMs were treated with GA (100 *μ*g/mL) for 48 h. Cells were harvested and stained with antibodies to CD80, CD86, and MHCII. Expressions of the surface molecules were analyzed by FACS and displayed, respectively, by the single parameter diagram. The values shown in the profiles were the gated % and the MFI. Results are representative of three independent experiments.

Activated macrophages are able to present antigen to T cells and induce an effective T cell response [28]. One of the defining characteristics of an efficient antigen-presenting cell is the expression of MHCII and costimulatory molecules like CD80 and CD86. To investigate whether GA regulates the expression of such molecules on macrophages, BMDMs were incubated with GA (100 *μ*g/mL) for 48 h. As shown in Figure 2, the percentage of CD80, CD86, and MHCII expression after GA treatment was upregulated from 9.86%, 90.84%, and 26.66% to 22.59%, 97.97%, and 63.16%, respectively. Meanwhile, the intensity of expression of all three molecules was significantly enhanced by the GA treatment. These data demonstrated that the BMDMs activation was effectively induced by the dose of GA (100 *μ*g/mL).

### 3.3. GA Promotes M1, rather than M2 Macrophages Polarization of BMDMs

**Figure 3:**
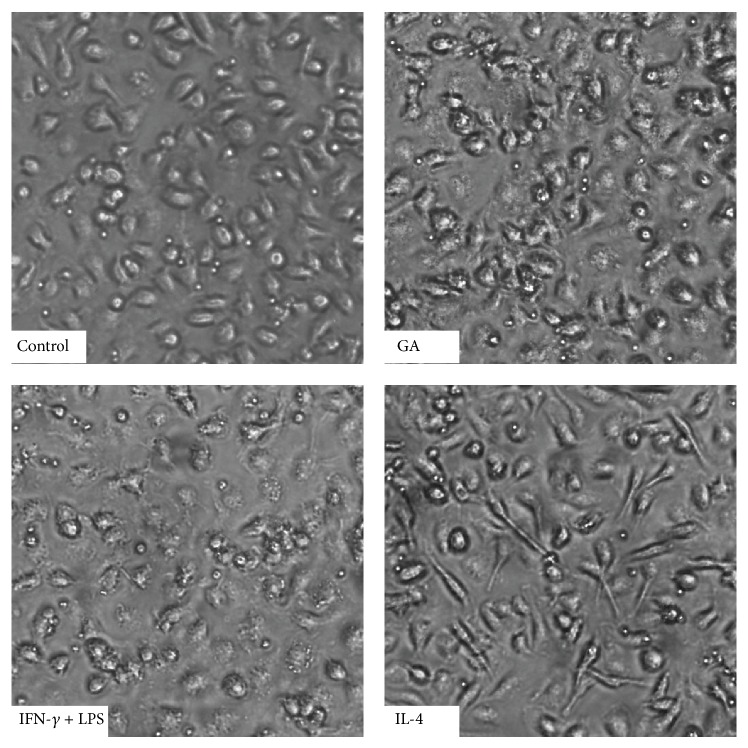
Effect of GA on morphological characteristics of BMDMs. BMDMs were treated with PBS, GA (100 *μ*g/mL), IFN-*γ* (15 ng/mL) + LPS (15 ng/mL), and IL-4 (20 ng/mL) for 48 h. The cell images were obtained under a light microscope (×200).

**Figure 4:**
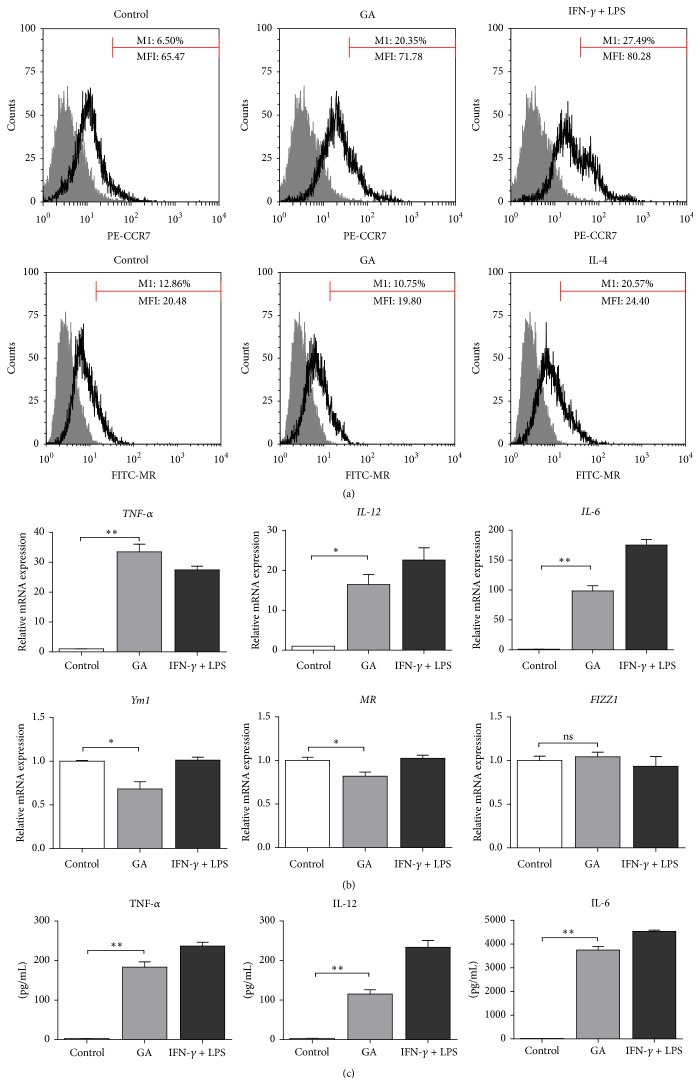
Effect of GA on M1- and M2-related markers expression in BMDMs. BMDMs were treated with GA (100 *μ*g/mL) for 48 h, the cells and supernatants were collected. (a) CCR7 and MR expressions were analyzed by FACS and (c) TNF-*α*, IL-12, and IL-6 secretions were quantified by ELISA. (b) BMDMs were treated with GA (100 *μ*g/mL) for 6 h, total RNA was extracted, and* TNF-α*,* IL-12*,* IL-6*,* Ym1*,* MR,* and* FIZZ1* expressions were measured by real-time PCR. Data are expressed as mean ± SD from three independent experiments. ^*∗*^ *P* < 0.05, ^*∗∗*^ *P* < 0.01 (*t*-test).

We further studied the effect of GA on BMDMs polarization. First, cell images were captured after 48 h of treatment. BMDMs in the control groups were oval or irregularly shaped cells with clear cell borders, while the GA-treated cells exhibited more pits and vacuoles and closely attached to the button with less distinct cell borders. As shown in Figure 3, the morphological characteristics of the GA-treated cells were more similar to those in M1-positive groups. To confirm whether GA mediates M1 macrophages polarization of BMDMs, we examined the expressions of M1- and M2-associated markers (Figure 4). FACS analysis demonstrated that GA markedly increased the expression of M1 surface marker CCR7, from 6.5% to 20.35%, whereas it decreased the expression of M2 surface marker MR, from 12.86% to 10.75% (Figure 4(a)). Furthermore, GA upregulated the expression of M1-associated cytokines, such as TNF-*α*, IL-12, and IL-6, at both the mRNA and protein levels; however, GA downregulated the mRNA expression of M2 markers, such as Ym1 and MR (Figures 4(b) and 4(c)). These results indicated that GA promotes typical M1 activation phenotype in BMDMs.

### 3.4. GA Induces NO Synthesis in BMDMs

**Figure 5:**
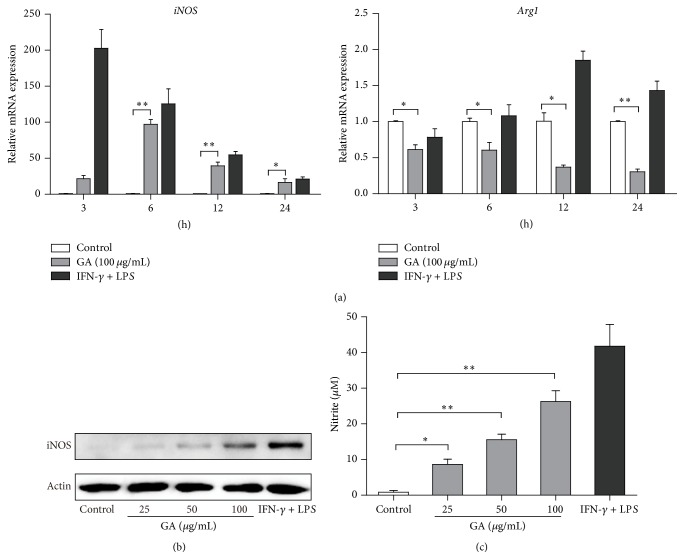
Effect of GA on NO synthesis in BMDMs. BMDMs were treated with GA (25~100 *μ*g/mL). (a) At the indicated time points, total RNA was extracted and the expressions of* iNOS* and* Arg1* were measured by real-time PCR. (b, c) After 24 h of treatment, cells and supernatants were collected, cell lysates were prepared and subjected to western blotting with iNOS and *β*-actin antibody, and the nitrite generation in the supernatants was quantified as described in Section 2. Data are mean ± SD for three independent experiments. ^*∗*^ *P* < 0.05, ^*∗∗*^ *P* < 0.01 (*t*-test).

M1 macrophages produce inducible nitric oxide synthase (iNOS) that enables the cell to kill intracellular pathogens through the production of NO, while M2 macrophages counteract iNOS activity by producing Arg1 which competes with iNOS for the same substrate, arginine, thus decreasing NO generation [29]. We further investigated whether GA regulates NO production in BMDMs. As shown in Figure 5(a), iNOS and Arg1 gene products were reciprocally regulated by the GA treatment, as follows: iNOS mRNA expression induced by GA was detectable by 3 h and increased to its peak at 6 h, whereas Arg1 mRNA was decreased by GA from basal levels to 30.64% of control at 24 h. As anticipated, we also observed that GA obviously enhanced iNOS protein expression in BMDMs in a dose-dependent manner (Figure 5(b)), and accordingly, the NO generation of GA-treated groups was significantly higher than those in the control groups (Figure 5(c)). In summary, these data supported the notion that GA could promote M1 macrophage polarization in BMDMs.

### 3.5. GA Enhances Phagocytosis and Bactericidal Capacity of BMDMs

**Figure 6:**
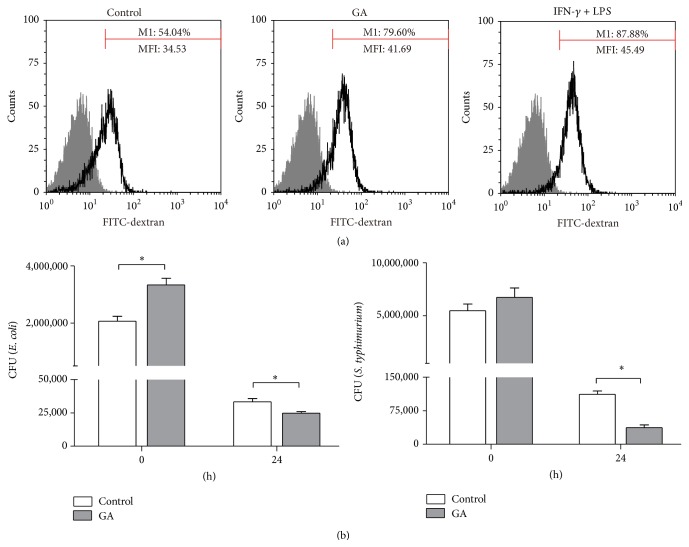
Effect of GA on BMDMs phagocytosis and bactericidal activity. BMDMs were pretreated with GA (100 *μ*g/mL) for 12 h. (a) Incubated with FITC-dextran at 37°C for 1 h, the intracellular FITC-dextran was measured by FACS. (b) After being infected with* E. coli* K88 or* S. typhimurium* for 1 h, washed, and incubated in DMEM with gentamicin (25 *μ*g/mL) for 0 h or 24 h, these cells were lysed, diluted, and plated on LB plates for colony enumeration. Data are mean ± SD for three independent experiments. ^*∗*^ *P* < 0.05 (*t*-test).

Phagocytosis and bacterial killing play a crucial role in macrophage-mediated host defense, which lead to internalization and destruction of pathogens. To determine whether GA triggers such functions on M1-polarized BMDMs, we first examined the internalization of FITC-labeled dextran by FACS. The BMDMs pretreated with GA for 12 h showed markedly increased uptake of FITC-dextran as compared with the control groups (Figure 6(a)). To support these findings, we evaluated the effect of GA on the uptake of* E. coli* K88 and* S. typhimurium* by BMDMs. Interestingly, GA significantly increased the uptake of extracellular bacteria* E. coli* K88 as compared with the control groups, while the internalization of intracellular bacteria* S. typhimurium* had no significant difference between the GA-treated groups and the control groups (Figure 6(b)). These inconsistent results of* E. coli* K88 and* S. typhimurium* internalization by BMDMs may result from the mechanisms by which* S. typhimurium* efficiently invades macrophages. After bacteria internalization by BMDMs, we further examined the bactericidal capacity of macrophages. As shown in Figure 6(b), GA markedly reduced the bacterial survival both in* E. coli* K88 and* S. typhimurium* infection models, relative to the control groups, respectively. To sum up, these results demonstrated that GA could enhance the phagocytic and bactericidal capacity of BMDMs.

### 3.6. GA Activates MAPKs and NF-*κ*B Pathways in BMDMs

**Figure 7:**
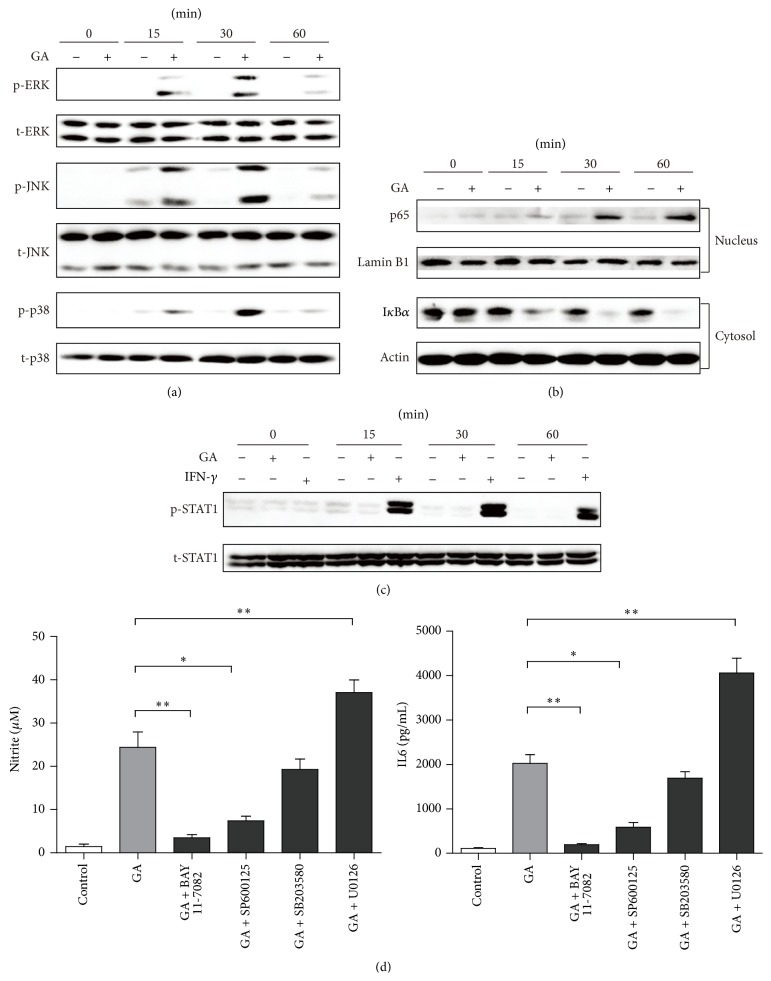
Effect of GA on MAPKs, NF-*κ*B, and STAT1 activation in BMDMs. BMDMs were treated with GA (100 *μ*g/mL) or IFN-*γ* (100 ng/mL) for the indicated time points. (a, c) Cell lysates were prepared, and phosphorylation of ERK1/2 (p-ERK1/2), JNK (p-JNK), p38 MAPK (p-p38), and STAT1 (p-STAT1) was analyzed by western blotting. (b) Nuclear and cytosolic extracts were prepared and subjected to western blotting with p65 and I*κ*B*α* antibody, respectively. (d) BMDMs were pretreated with NF-*κ*B inhibitor (BAY 11-7082, 10 *μ*M), JNK inhibitor (SP600125, 10 *μ*M), p38 MAPK inhibitor (SB203580, 10 *μ*M), or ERK1/2 inhibitor (U0126, 10 *μ*M) followed by treatment with GA (100 *μ*g/mL) for 48 h, and then the production of nitrite and M1-related cytokines was analyzed. Data are mean ± SD for three independent experiments. ^*∗*^ *P* < 0.05, ^*∗∗*^ *P* < 0.01 (*t*-test).

Macrophage polarization is a complex process including stimuli recognition and activation of the transcription factors [30]. Recent studies have shown that MAPKs, NF-*κ*B, and STAT1 signaling pathways are involved in M1 macrophage polarization [16, 31]. To investigate whether GA activates these cascades, we performed western blotting to examine the phosphorylation of MAPKs and STAT1 and the nuclear translocation of NF-*κ*B p65. Treatment with GA led to a rapid and transient increase in the phosphorylated forms of all three MAPKs in BMDMs, and phosphorylation reached its peak at 30 min of treatment and declined to almost basal level within 60 min (Figure 7(a)). We also observed that GA triggered a gradual increase of NF-*κ*B p65 protein in the nucleus, while it correspondingly decreased I*κ*B*α* protein in the cytosol within 60 min of stimulation (Figure 7(b)). However, GA did not induce phosphorylation of STAT1 in BMDMs (Figure 7(c)), indicating that GA-mediated M1 macrophage polarization was independent of STAT1 signaling.

### 3.7. GA-Induced M1 Macrophages Polarization Is Mediated by JNK and NF-*κ*B

**Figure 8:**
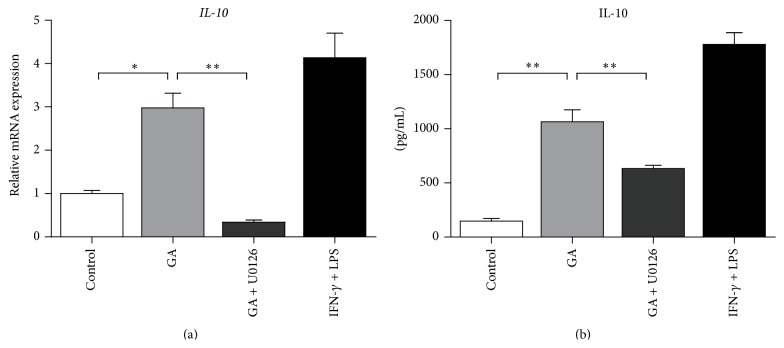
Effect of ERK1/2 pathway on GA-induced IL-10 production in M1-polarized BMDMs. BMDMs were pretreated with ERK1/2 inhibitor (U0126, 10 *μ*M) followed by treatment with GA (100 *μ*g/mL) for (a) 6 h or (b) 48 h, and* IL-10* mRNA expression and IL-10 secretion were measured by real-time PCR and ELISA, respectively. Data are mean ± SD for three independent experiments. ^*∗*^ *P* < 0.05, ^*∗∗*^ *P* < 0.01 (*t*-test).

We next determine the role of MAPKs and NF-*κ*B activation in GA-induced M1 macrophages polarization. BMDMs were, respectively, pretreated with pharmacological inhibitors for 30 min and then incubated with GA for 48 h. As shown in Figure 7(d), Bay 11-0782 (NF-*κ*B) and SP600125 (JNK), but not SB203580 (p38 MAPK), significantly decreased GA-induced production of NO and M1-related cytokines (shown with IL-6 as an example), whereas pretreatment with U0126 (ERK1/2) resulted in unexpected increases in both NO and M1 cytokines production. Furthermore, inhibition of ERK1/2 pathway led to a marked decrease of IL-10 expression, at both the mRNA and protein levels (Figure 8). Together, these data thus indicated that NF-*κ*B and JNK activation are required for GA-induced M1 macrophages polarization, while ERK1/2 pathway exhibits a regulatory effect via induction of inhibitory factors, such as IL-10.

## 4. Discussion

Macrophages are an extremely heterogeneous lineage displaying a range of both pro- and anti-inflammatory functions. A functional phenotype obtained by macrophages is dependent upon interactions with specific stimuli from both endogenous and exogenous environment [17]. However, unlike T cells, which undergo extensive epigenetic modifications during differentiation, macrophages seem to retain their plasticity and respond to further environmental signals [16]. Previous studies observed that a phenotypic switch from M1 to M2 phenotype in the macrophage population occurs in the pathological process of cancer [16, 30]. Moreover, some pathogens, such as* Francisella tularensis* and* Leishmania major*, are able to redirect the phenotype of macrophages from M1 to M2 and consequently survive at the expense of the host [29, 32]. Therefore, targeting of this macrophage phenotypic plasticity may provide a novel therapeutic strategy for such diseases and infections.

Here, we investigated the effect of GA on the phenotypic polarization of macrophages. Our data showed that GA markedly increased the expression of CD80, CD86, and MHCII molecules, which associated with the antigen presentation and T lymphocyte activation. Similar results were obtained from GA-treated DCs that induced Th1 responses [10]. We further analyzed the expression of M1- and M2-related markers in BMDMs and found that GA significantly upregulated the expression of CCR7, TNF-*α*, IL12, and IL-6, which are related to M1 macrophages, whereas GA downregulated the expression of M2-related markers MR and Ym1. These results are in agreement with previous studies that GA exhibited a stimulatory effect on coronavirus and* Leishmania donovani* infected peritoneal macrophages via induction of proinflammatory effectors [8, 33].

The NO metabolism is an important distinction between M1 and M2 macrophages. M1 macrophages produce high levels of iNOS which synthesizes NO from arginine, while M2 macrophages counteract iNOS activity by producing Arg1 which degrades arginine to urea and ornithine, thus reducing the production of NO [29]. We observed that GA reciprocally regulated the expression of iNOS and Arg1 in BMDMs, which led to an increased production of NO. These results are supported by a report that GA enhanced NO production in IFN-*γ*-activated macrophages [14]. Moreover, M1 macrophages are known as potent effector cells with enhanced phagocytosis and bactericidal capacity [16]. Consistent with these notions, GA enhanced uptake of FITC-dextran and* E. coli* K88 by BMDMs and markedly decreased the intracellular survival of* E. coli* K88 and* S. typhimurium*. To the best of our knowledge, there is no previous report on the bacterial killing capacity of GA-treated macrophages. Our data provide evidence that GA exhibits a protective effect in host defense against intracellular microorganisms by mediating the M1 functional polarization of macrophages.

Since a macrophage polarization requires activation of specific transcription factors, the possible pathways involved in GA-polarized M1 macrophages could be carefully identified. It is known that MAPKs (ERK, JNK, and p38 MAPK) regulate various inflammatory cytokines expression through phosphorylation of transcription factors. NF-*κ*B proteins are detached from its inhibitor I*κ*Bs after activation and translocate from the cytoplasm to the nucleus and finally regulate the transcription of a large number of genes [34]. We found that GA induced phosphorylation of all three MAPKs, which was accompanied by nuclear translocation of NF-*κ*B p65. Inhibition of JNK and NF-*κ*B activation by their respective inhibitors significantly decreased GA-induced production of NO and M1-related cytokines. Recent study demonstrated that JNK was required for M1 macrophage polarization in HFD-fed mice, and deficiency of JNK led to decreased expression of M1 genes and increased expression of M2 genes in adipose tissue macrophages [31]. In addition, NF-*κ*B pathway was also reported to be associated with M1 macrophage polarization [16]. Inhibition of p38 MAPK did no effect on GA-induced M1 markers; however, inhibition of ERK1/2 resulted in unexpected increases in both NO and M1 cytokines production. ERK1/2 seems to be a regulator which limits inflammation via induction of anti-inflammatory cytokines [35]. We further confirmed that inhibition of ERK1/2 markedly decreased GA-induced IL-10 expression in BMDMs. To sum up, JNK and NF-*κ*B activation are required for GA-induced M1 macrophages polarization, while ERK1/2 pathway exhibits a regulatory effect in prevention of excessive inflammation.

It should also be noted that some studies reported that GA inhibited the induction of proinflammatory effectors induced by TLR (toll-like receptor) agonists (e.g., CpG-DNA and LPS). In fact, the anti-inflammatory activity of GA may result from the changes in cell membrane, thus attenuating membrane-dependent receptor signaling. For instance, GA decreased cellular attachment or uptake of CpG-DNA and strongly impaired LPS-induced homodimerization of TLR4 in RAW 264.7 cells [16, 36, 37]. Here, we demonstrated that GA itself exhibits stimulatory effect, rather than inhibitory effect on BMDMs by skewing the M1 macrophage polarization bias.

It is still unclear how macrophages recognize GA, but the interaction between GA and cell membrane has been observed [36–38]. Our data showed a clear relationship between GA and M1 macrophage polarization which is known to be important for the clearance of intracellular pathogens and tumor cells. As the phenotype of a macrophage population appears to be changed by specific stimuli [16], GA might be a useful therapeutic for M2-associated diseases or infections by converting the macrophage population from M2 to M1 phenotype. Hence, it would be interesting to further introduce* in vivo* and* in vitro* studies to explore the role of GA in M2-dominated models.

## 5. Conclusion

In conclusion, we demonstrated a novel role for GA in the polarization of M1 macrophage. These findings might give a new insight into the function of GA on immune system and highlight the clinical significance of GA as a positive modulator in response to pathogens invasion.
